# Exploring the Use of a Qualitative Behavioural Assessment Approach to Assess Emotional State of Calves in Rodeos

**DOI:** 10.3390/ani10010113

**Published:** 2020-01-10

**Authors:** Sally Rizzuto, Di Evans, Bethany Wilson, Paul McGreevy

**Affiliations:** 1 School of Veterinary Science, University of Sydney, Camden, New South Wales 2570, Australia; 2 Science Team, RSPCA Australia, Deakin, ACT 2600, Australia; devans@rspca.org.au; 3 School of Veterinary Science, University of Sydney, Camperdown, New South Wales 2006, Australia; bethany.wilson@sydney.edu.au (B.W.); paul.mcgreevy@sydney.edu.au (P.M.)

**Keywords:** qualitative behavioural assessment, calf, calf-roping, rope-and-tie, rodeo, animal behaviour, behavioural assessment, animal wellbeing

## Abstract

**Simple Summary:**

The nature of rodeo events, including calf-roping, bull- and bronco-riding and steer-wrestling, regularly subject animals to rough handling in ways likely to elicit fear. However, there have been only limited attempts to assess the emotional state of animals undergoing these handling techniques. The current project aims to explore the potential of a qualitative behavioural assessment (QBA) approach to estimate various emotional states by examining images of calves before and after being roped during a rodeo event. QBA is based on human descriptors that summarise the dynamic, expressive style of an animal’s interaction with its environment; e.g., relieved, calm, contented, or anxious. The study found that calves show very different emotions (what they are experiencing emotionally) and behaviours (what they are doing) during the two phases of calf-roping assessed: the chase and recovery. These results indicate that a QBA approach has potential for assessing animal welfare in the entertainment industry.

**Abstract:**

There are longstanding disagreements between the rodeo industry stakeholders and animal welfare advocates about the wellbeing of the animals used in events. The current study aims to determine whether qualitative behavioural assessment (QBA) is effective in identifying the emotional state of calves in so-called calf-roping events. Still images of calves captured from videos of calf-roping were shown to two groups: practitioners (*n* = 7) and students (*n* = 16). For each image, they scored (on a scale of 1–10) 12 descriptive terms—e.g., stressed, energetic, confused, frightened—based on how strongly they thought the animal was experiencing that emotion. Scores were analysed using Factor Analysis and Ordinal Logistic Regression models, while inter-rater reliability was assessed using Intra-Class Correlation Coefficients. The same imagery (video and images) were analysed for behaviours associated with the calves’ ears, neck, legs and tail to develop a behavioural ethogram, which was analysed with Binary Logistic Regression and Anova wrapping. The students were also surveyed to assess their empathy towards animals. The chase phase attracted significantly higher scores for stressed (µ = 5.0, *p* < 0.001), agitated (µ = 5.1, *p* < 0.001), anxious (µ = 5.0, *p* < 0.001) and frightened (µ = 5.0, *p* < 0.001), and the behavioural ethogram revealed that calves commonly galloped (*p* < 0.001) and held their tails rigidly during this phase (*p* = 0.010). In contrast, the recovery phase was characterised by significantly higher scores for calm (µ = 3.0, *p* < 0.001), contented (µ = 2.7, *p* < 0.001) and relieved (µ = 1.6, *p* < 0.001), and calves moved slower (*p* < 0.001) with more neutral ear positions (ears axial *p* = 0.008, ears forward *p* = 0.010). A clear pre- and post-rope effect was evident, showing that QBA indicated that calves were anxious while being chased and were relieved when they had been released. The survey data revealed that students who had more empathy for animals in pain and for those used in experiments were more empathetic towards calves during the chase phase. They felt that calves being chased were agitated, anxious, stressed, frightened and confused. These results confirm that QBA has the potential as a tool for assessing the welfare of animals used in rodeos.

## 1. Introduction

Rodeos originated from cattle-ranching culture and were initially used to hone and display the everyday practical skills that stockpersons needed to run a successful ranch [1]. In particular, calf-roping was used on farms to facilitate the performance of routine veterinary procedures, such as castration and dehorning. With the progressive transition from horse-backed ranching to motor-vehicle-based ranching, rodeos developed into the current entertainment event [1]. This means that currently the chief motivations for running rodeo events are economic gains, entertainment and competitive sportsmanship rather than the practical application of skills.

**Figure 1:**
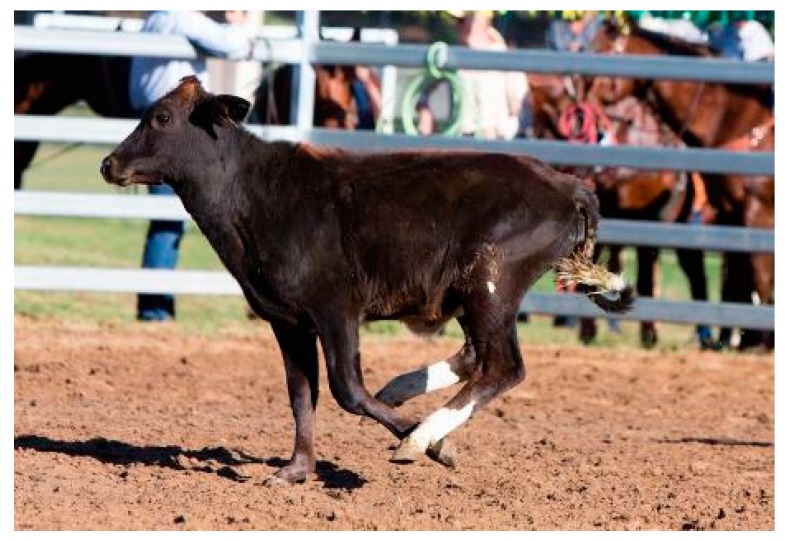
Typical ‘chase’ image within one second of being released from the chute.

**Figure 2:**
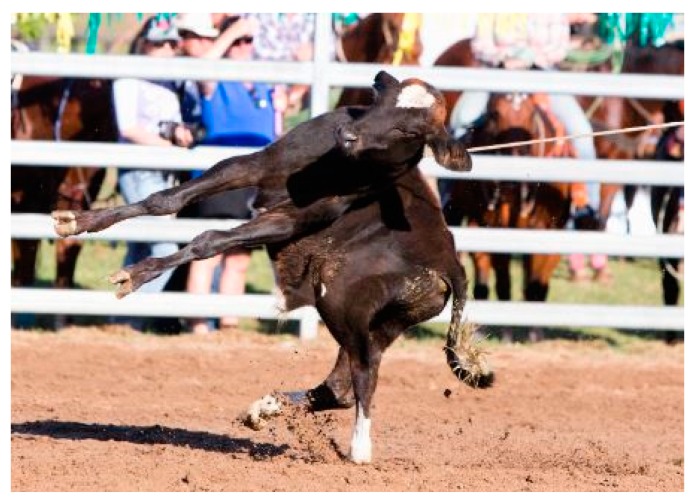
Typical image of calf being lassoed.

Given the current focus on entertaining audiences, rodeos are subject to criticism for their alleged disregard for animal welfare [1,2]. In Australia, the United States of America, and Canada, the six most common rodeo events are saddle bronc-riding, bareback-bronc riding, bull-riding, steer-wrestling, team roping, and rope-and-tie (calf-roping) [3]. Of these events, calf-roping is perhaps subject to the most scrutiny from concerned observers. It involves a calf being released from a barrier or chute and chased by a competitor on horseback. The rider’s goal is to rope the calf, dismount, then run to and throw the calf onto their side in order to tie three of their legs together [3]. The competitor’s time is recorded at the completion of the tie. The rider must then remount and release the tension on the rope to show that the tie holds [3]. The winner is the competitor with the fastest time and a secure tie. The Figure 1 and Figure 2 depict different stages of calf-roping.

Animal advocates have voiced several concerns about calf-roping. These include the prospect that calves can suffer physical injuries when roped and thrown to the ground, including broken bones and internal injuries such as haemorrhage and tracheal damage, some of which may necessitate euthanasia [4]. Furthermore, animals are subject to fear-eliciting scenarios that elevate levels of stress and suffering [5]. In their defence, the rodeo industry and related stakeholders maintain that rodeo animals are treated humanely and with dignity, are protected in Australia under the Code of Conduct for the Welfare of Rodeo Livestock and have a low injury rate [6]. The rodeo industry states that “one injury is recorded for every 3471 times an animal is used [and] one animal is severely injured or euthanised for every 5571 times an animal is used” [6]. Competition rules for calf-roping include disqualification and fines for ill-treating stock, severe jerking or throwing animals to the ground and if the horse drags the calf more than 1 metre [7]. Also, an approved roping device [8], which is designed to work as a shock absorber and thus reduce the force of the rope around the animal’s neck, must always be used under industry rules [7]. It is also important to note that despite the colloquial name for this event being “calf-roping”, its official name is the Rope and Tie. Animals must be more than 100 kg, have been weaned and be at least 12 weeks old [7,9]. Nevertheless, for the purposes of contextualization, the current article will refer to these animals as calves. A minimum body weight limit of 200 kg for cattle has been set in Victoria and South Australia, effectively prohibiting calf-roping, as the maximum weight stipulated by at least one rodeo association is 140 kg, which is much less than 200 kg. Furthermore, weaned animals who are at least 200 kg would no longer be considered calves, and anecdotal reports indicate that it is too dangerous for the “calf” and the competitor to rope-and-tie an animal weighing over 200 kg, thus setting the minimum body weight limit of 200 kg for participating bovids effectively prohibits calves being roped.

Despite the history of conflicting views from the rodeo industry and animal welfare advocates, there is little research on the physiological, behavioural and emotional responses of animals participating in rodeo events. The limited published research focuses primarily on bulls and calves used in bull-riding and calf-roping [1,2,10] and has reported contradicting results.

A study of bull behaviour at rodeos [2] determined that 30% of 14 animals showed signs of distress leading up to the start of bull-riding events, which the authors reported as being low.

Physiological responses in the bulls were not reported in this study. In contrast, calves have been found to show physiological stress responses during calf-roping, irrespective of whether they had been roped before [1].

Another study [10] explored the short-term welfare of calves (*n* = 30) roped at a rodeo. Changes in creatinine kinase, lactate hydrogenase enzymes and cortisol concentrations in calves that were roped were compared to a control group of calves that were present at the rodeo but did not take part in the roping event. Head-shaking and sham-chewing were evident in a third of the calves after they had been roped. There were significant increases in the concentrations of creatinine kinase, lactate dehydrogenase enzymes and cortisol in roped calves. However, these increases could not be attributed solely to being roped, thrown and tied because significant increases were found in calves who had been pursued by riders but not roped successfully.

The dearth of empirical data on perceived emotional states among animals participating in rodeos contributes to the difficulty faced by governing bodies when they attempt to make well-informed decisions about animal welfare. However, a unique research opportunity to address this knowledge gap recently arose with the introduction of new animal welfare legislation [11] in Quebec, Canada, in 2015. According to this legislation, a person may not “cause an animal to be in distress” unless it is for agriculture, teaching, science or veterinary treatment, and is justified, minimal or cannot be alleviated. Although permitted use includes agricultural exhibitions and fairs, the legislation essentially excludes animals used for entertainment such as rodeos and circuses. This possible conflict led to researchers being granted access to rodeos to gather material to assess the welfare of the animals involved.

There is a growing body of work that supports the feasibility of using qualitative behavioural assessment (QBA) to assess the emotional state of animals. QBA is a method that involves observers viewing images and/or footage and then scoring, on a scale between a minimum and maximum score, using a series of descriptive terms according to how well they describe the emotional state of the animals in the images. Scores are analysed to determine common patterns between animals, treatment groups and individual observers [12].

Historically, QBA has been successfully applied to assess the behaviour of dairy cows with clinical mastitis [13], during stockperson interaction [14] and in housing systems [15], and beef cattle during transport [16], transport and lairage [17], castration [18] and welfare assessments [12,19]. Other livestock applications include the assessment of behaviour and flock health in sheep [20], and behaviour, emotionality and body language in pigs [21,22,23]. QBA has also been applied to the behaviour assessment of horses during competition [24] and affiliative and aversive human interactions [25,26], and even as an assessment tool for dogs entering shelters [27,28]. This extended body of published research suggests that QBA may be a reliable measure of the welfare of animals used in rodeos.

To our knowledge, there is no published study of audiences’ perceptions of animal use in rodeos. This knowledge gap may reflect the audience’s physical distance from the animals during rodeo events, a general lack of understanding of animal behaviour, disinterest in animal welfare or the overwhelming atmosphere of rodeos distracting viewers from watching individual animals closely. There is evidence that observers with relatively high empathy towards humans are more sensitive to indicators of cattle pain than less empathic observers [29]. One landmark study surveyed veterinary science students and gathered information on gender, year of study, demographics and views on animal sentience using an attitude to animals questionnaire [30]. This showed that gender and level of animal education are associated with perceived sentience in animals and emotional empathy towards them. Students in later years of study rated animals as having lower levels of sentience than students in earlier years and, throughout their studies, female students showed higher levels of empathy towards animals than male students did.

The objectives of the current study were to explore the feasibility of using QBA to characterise the behaviour of calves in still images from video footage taken at two different stages of the calf-roping event, and to identify if their behaviour varied between these stages. We hypothesised that QBA can be used to estimate different emotional states in calves as positive, negative or neutral as well as the intensity of what animals are experiencing emotionally before and after roping. Furthermore, the study explored whether observers’ scores for intensity of calves’ emotional state correlated to positive attitudes to animal sentience.

## 2. Materials and Methods

Approval for these procedures was granted by The University of Sydney Human Research Ethics Committee (Approval number: 2018/902).

### 2.1. Data Collection

#### 2.1.1. Image Set-Up and Recruitment

Footage of a calf-roping event was sourced from The Festival Western de St-Tite in Quebec, Canada during 9–17 September 2017. It was not feasible to obscure rodeo-specific elements in these videos, such as background features, ropes, rodeo horses and competitors to avoid bias among observers that may reflect their established beliefs about rodeos. Therefore, still images were isolated from video footage of a total of 55 calves at four different moments during the calf-roping event. These were screened according to inclusion and exclusion criteria that selected images of 20 calves. The first image (Chase A) was the first usable frame after the calf left the chute, and the second image (Chase B) was the next closest frame within 1 s of the first image. No images of calves actually being roped were used. The third image (Recovery C) was the first usable frame after the calf stood up after being roped, tied and released, and the fourth image (Recovery D) was the next closest frame within 1 s of the third image. Each calf acted as their own control, with chase images (A and B) being the treatment (the aversive stimuli associated with being chased) and recovery images (C and D) being the comparison stage with the aversive stimuli no longer being applied.

Observers were recruited for the two rounds of data collection. For Round One, practitioner observers were recruited from veterinary graduates with practitioner expertise in calf behaviour, and agricultural and university lecturers involved in providing instruction on cattle handling (*n* = 7), whilst for Round Two, observers were drawn from volunteers among the Doctor of Veterinary Medicine and Animal & Veterinary Bioscience students from the University of Sydney (*n* = 16).

After isolating usable frames from the video footage, still images of 10 calves (images *n* = 40) were selected for practitioners to identify their preferred descriptors and compare them with the selected fixed-term descriptors (FTDs) used by the student observers.

An additional 10 calves (images *n* = 40) were chosen for the student observers to view in Round Two. These images were also used to develop a list of 12 putative FTDs of body language and facial expression. These descriptors were derived from the EU Welfare Quality project, which has been previously used in published studies of calf behavior [12]. These included agitated, anxious, confused, frightened, stressed, energetic, excited, inquisitive, calm, contented, relieved and exhausted. All images were edited to obscure rodeo-specific elements, such as background features, ropes, rodeo horses and competitors. 

#### 2.1.2. Validation of Fixed-Term Descriptors

For Round One, the first 40 images and 12 FTDs were randomised and loaded into an online survey program (Qualtrics, Provo, UT). Each practitioner received an online Participant Information Sheet outlining the study and their role, and an online training document containing instructions, example questions and the link to the survey. Of the eight practitioners who were invited, seven participated. The survey presented one image at a time and asked participants to score the image using the 12 FTDs on a scale of 0–10 based on how strongly they felt the animal was experiencing each particular emotional state. A score of 0/10 meant they did not think the animal fitted the descriptive term at all, whereas a score of 10/10 meant they thought the animal fitted the descriptive term very strongly. After assessing all 40 images, the practitioners were invited to suggest additions to, removals from or substitutions for the list of FTDs.

#### 2.1.3. Assessment of Emotional State

For Round Two, the survey was duplicated with 40 images added at random (*n* = 80). Observers received a Participant Information Sheet and the same training document (as above). The survey format and instructions were also identical to Round One, except for the total number of images. Footage of sufficient quality for two calves for Round Two could not be isolated, which necessitated the re-use of two calves’ footage; i.e., two calves appeared twice in Round Two, although different frames were selected.

For both rounds, the context of calf-roping was not disclosed to practitioners (except one observer, who was notified because of an administration error) or student observers and all results were anonymous. Data were collated and exported from the online platform and received by a statistician who was blinded to the phase of the images, appearance of images, and identities or level of personal experience of the recruited participants beyond whether they were in the practitioner or student cohorts.

#### 2.1.4. Empathy towards Animals (ETA) Survey

Student observers were asked to complete an online questionnaire about their demographics, attitudes towards animals with a focus on animal welfare and sentience. Part 1 and 4 asked questions about age, gender, demographics and course being studied. Part 2 was associated with gauging views towards animal sentience, and Part 3 tested empathy towards animals. Questions in Part 3 were based on Paul and Podberscek’s [30] Animal Empathy Scale. The online questionnaire can be found in [31].

#### 2.1.5. Ethogram

The video footage of the calves from which the still images were derived was used to develop a behavioural ethogram. Behaviours indicated by changes in ears, nose, neck, legs and tails were tallied as absent or present in each chase and recovery phase. The behaviours coded as present/absent were as follows: ears held axial; ears held forward; ears held backward and upward; ears held backward and downward; nostrils flaring; neck held horizontal; neck held above horizontal; neck held below horizontal; legs at a gallop; legs at a slow run; legs at a fast walk; tail wagging with a rigid motion; tail wagging with a vigorous motion; tail tucked behind; tail erect and rigidly held.

### 2.2. Statistical Analysis

#### 2.2.1. Assessment of Emotional State Data

Factor analysis was employed to explore the Round One data and, for comparison, the Round Two observer data were used to explore the underlying structure of the FTDs scores. The number of factors of interest was decided by visual identification of the elbow in the Scree plot and by use of the “nScree” function of the nFactors package of R Statistical Software [32] which uses the Eigenvalues (the “Kaiser rule”) and parallel analysis.

A maximum likelihood factor analysis was then performed, using the “factanal” function from the statistics package to fit the selected number of factors following a varimax rotation to increase the interpretability of the extracted factors.

Where possible, scores from the Round Two observers were analysed by ordinal logistic regression. Analysis of the 11 rank scores as a continuously varying trait was considered. However, in many instances, the skewness and kurtosis of scores did not resemble a normal distribution, raising concern about the eventual distribution of residuals. Ordinal logistic regression allowed leveraging of the order of the rank scores without making assumptions of normality.

The ordinal logistic regression model used was a conditional proportional log odds model:

$$\mathrm{logit}\left[P\left(Y\leq j\right)\right]={\;\alpha }_{j}+x_{i}\beta^{\prime },\;j=1,\;2\ldots ..10$$

where each threshold, j, had its own intercept α_j_, with the constraint that α_1_ < α_2_ < α_….._ < α_10_. *β*’ is a vector of fixed effects related to a vector of explanatory variables (x_i_) and the model was fitted using the “polr” function of the Modern Applied Statistics with S package for R statistical software. The t value produced was compared to the normal distribution, using the function “pnorm”.

Explanatory variables included a fixed effect identifying the calf, a fixed effect identifying whether this was the first or second use of this calf’s image, a fixed effect timing of the image (Chase A, Chase B, Recovery C or Recovery D, as described above) and a fixed effect identifying the Round Two observer providing the score.

Inter-rater reliability was assessed by calculating the intra-class correlation coefficient for the consistency of the different raters’ scores from both Round One data and Round Two. For the Round One data, a two-way mixed effect model was employed, whereas for the Round Two data a two-way random effect model was employed. The “icc” function from the irr package was employed. The practitioner observers were individuals who had been approached primarily because of their expertise in cattle handling, welfare or behavior; the students were sourced from a wider pool of students, any of whom might have opted to complete the survey. The variability of their responses was expected to be higher than that of the practitioners’ responses.

#### 2.2.2. ETA Survey

To facilitate the analysis of associations between the FTD scores and ETA scores of the students, the QBA scores of the students were reduced from to a single dimension summarising their scores using two methodologies.

(1) Firstly, to examine the absolute magnitude of emotion scored (in Chase A), the unrotated First Principal Component Factor scores were used. This is equivalent to the most informative linear combination. Principal Component Factor 1 scores for Chase A were assessed using a linear mixed effect model:
  PC1 ~ age of student + gender of student + student response to an ETA item + random individual calf effect + random individual student effect
  where PC1 corresponds to the unrotated First Principal Component Factor score of a student’s QBA score for a photo; the age of the student is a fixed effect; the gender of the student is a fixed effect; and student response to the ETA item is a fixed effect of the response given by the student to an ETA item. Random effects for individual calf effects and individual student effects on the PC1 score were also included.
(2) Secondly, to look at the change in emotions perceived in the change from the Chase to the Recovery phase, the differences between scores for Chase B and Recovery C from each observer were summed for each of the FTDs. So, observers who detected more change between Chase B and Recovery C had a higher overall score for that FTD, while those who scored Chase B and Recovery C similarly had lower scores. These delta scores between Chase B and Recovery C were assessed using a linear mixed effect model.
  Total_behaviour_delta~ age of student + gender of student + student response to an ETA item + random individual calf effect + random individual Student effect
  where Total-behaviour_delta corresponds to the total difference between the student’s QBA score for a photo for each FTD; the age of the student is a fixed effect; the gender of the student is a fixed effect; and student response to the ETA item is a fixed effect of the response given by the student to an ETA item. Random effects for individual calf effects and individual student effects on the Total-behaviour_delta score were also included.

#### 2.2.3. Ethogram

The data collected from the ethogram were assessed by binary logistic regression using the glm function of the stats package of R, then applying an ANOVA wrapping using the “Anova” function of the car package.

## 3. Results

### 3.1. Factors Contributing to Responses

**Figure 3:**
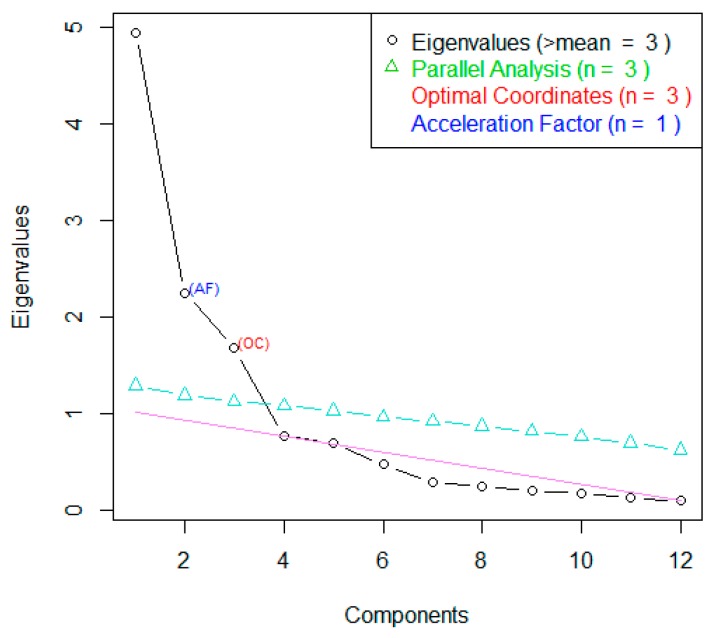
Factor loadings of Factor Analysis of Fixed Term Descriptors applied by a panel of seven practitioners on 40 images of calves (*n* = 10) during a calf roping event.

Factor Analysis and Varimax rotation were conducted on the 12 FTDs from Round One. The Scree Plot (Figure 3) showed points of inflexion after the fourth and third factor, with Factors 1–3 having an eigenvalue > 1. Cumulative variance showed that the first four factors explained 72% of the variance in this dataset. This suggests three-to-four potentially informative factors.

Factor 1 strongly loaded agitated, anxious, frightened and stressed, suggesting it represented negative emotions (Table 1). Factor 2 strongly loaded energetic and excited, suggesting it represented positive emotions. Factor 3 strongly loaded calm and contented, suggesting it represented neutral emotions.

**Figure 4:**
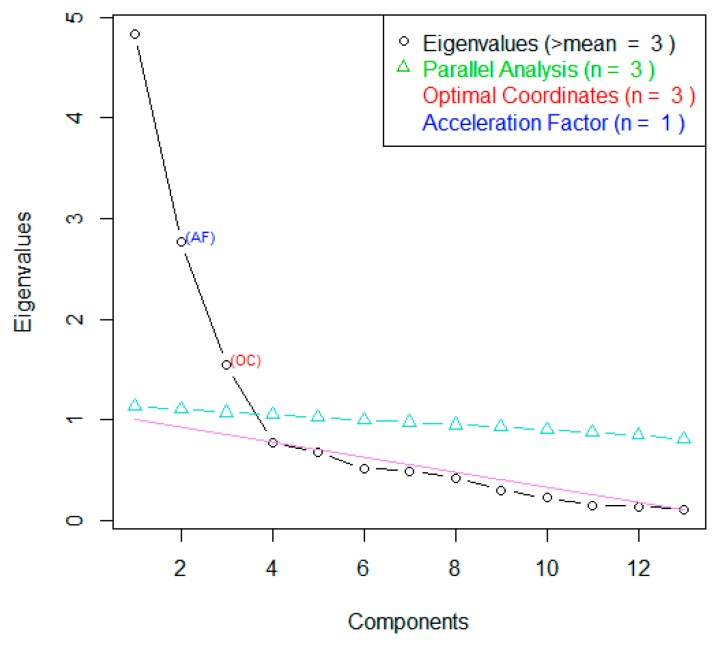
Scree Plot for Factor Analysis of Fixed Term Descriptors applied by a panel of 16 students on 80 images of calves (*n* = 20) during a calf roping event.

Similarly, Factor Analysis and Varimax rotation of the 12 FTDs were carried out on the Round Two responses. The Scree Plot (Figure 4) showed points of inflexion after the fourth and third factors, with Factors 1–3 having an eigenvalue > 1. Cumulative variance showed that the first four factors explained 69% of the variance in this dataset. Once again, this suggested three-to-four potentially informative factors.

Factor loadings for Round Two were broadly similar to Round One. Factor 1 strongly loaded agitated, anxious, frightened and stressed, again suggesting it represented negative emotions (Table 2). Factor 2 strongly loaded calm and contented, suggesting it represented neutral emotions and is similar to Factor 3 in the Round One loadings. Factor 3 strongly loaded energetic and excited, suggesting it represented positive emotions and resembled Factor 2 in the Round One loadings.

The underlying factors for Round Two differed between the chase and recovery phases (Table 3). Round Two scores for Factors 1–4 were significantly different for the recovery phase when compared to Chase A. Scores for Factor 3 in Chase B were also significantly different from those in Chase A.

### 3.2. Chase versus Recovery Phases and Intra-Phase Similarity

Mean scores for chase and recovery phases were calculated from the Round Two dataset. Compared with those for the recovery phase, the scores for the chase phase were higher for agitated (µ = 5.1), anxious (µ = 5.0), confused (µ = 3.0), energetic (µ = 4.3), excited (µ = 2.9), frightened (µ = 5.0) and stressed (µ = 5.1). In contrast, the recovery phase scored higher for calm (µ = 3.0), contented (µ = 2.7), exhausted (µ = 2.3), inquisitive (µ = 2.5) and relieved (µ = 1.6).

**Figure 5:**
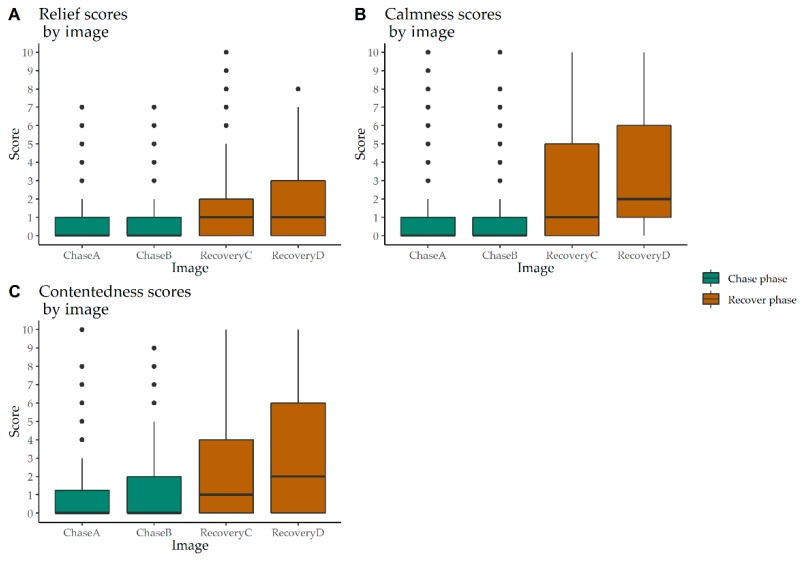
Box and whisker plot of relieved, calm and contented scores applied by a panel of 16 students on 80 images of calves (*n* = 20) showing higher scores in the recovery phase compared to the chase phase of a calf roping event. Outliers were present mostly in the chase phase.

**Figure 6:**
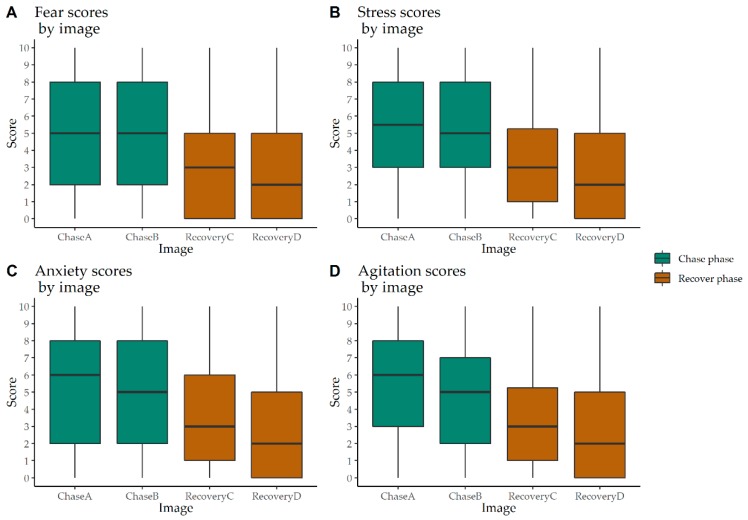
Box and whisker plot of frightened, stressed, anxious and agitated scores applied by a panel of 16 veterinary students on 80 images of calves (*n* = 20) showing higher scores in the chase phase compared to the recovery phase of a calf roping event.

Ordinal Logistic Regression was conducted on the 12 FTDs from Round Two, comparing Chase A to Chase B, Recovery C and Recovery D. A strong recovery effect emerged for relieved, calm, contented, frightened, stressed, anxious, and agitated (Figure 5 and Figure 6).

Scores for relieved were significantly higher (*p* < 0.001) during both recovery phases than in Chase A. Likewise, calm and contented scores were also significantly higher (*p* < 0.001) in both recovery phases. Scores between chase phases for these three FTDs were not significantly different. For frightened and stressed, both recovery phases scored significantly lower (*p* < 0.001) than the chase phases. Likewise, anxious and agitated scored significantly lower (*p* < 0.001) in both recovery phases than the chase phases. However, in the chase phase, scores among these four FTDs were not significantly different.

Recovery scores for inquisitive were significantly higher (*p* < 0.001) than Chase A, but so too were Chase B scores (*p* = 0.049). Recovery scores for energetic were significantly lower (*p* < 0.001) than Chase A, and Chase B were significantly higher (*p* = 0.0498). Recovery scores for confused were lower than Chase A but this was attributed chiefly to scores in Recovery D (*p* = 0.001). Scores between Chase A and Chase B were not significantly different. Recovery scores for excited were also lower than in Chase A, but this was significant for only Recovery C (*p* < 0.001). Chase B excited scores were also significantly higher than Chase A (*p* < 0.001). Recovery scores for exhausted were higher than Chase A, but this was significant only for Recovery D (*p* = 0.023). Scores between Chase phases were not significantly different. Unfortunately, due to the limited number of observers, it was not possible to compare responses within the student group by their attitudes to animals. Future studies to explore this relationship would benefit by having a sufficient number of observers.

### 3.3. Reliability of Scoring

Intra-Class Correlation Coefficients (ICCs) were calculated (Table 4) to measure the reliability of scoring within and between Round One and Round Two. ICCs for Round One were lower than for Round Two and, in some cases, not significantly different from zero.

For Round One, ICCs ranged from −0.00775 (confused) to 0.306 (calm) with reliability being highest (ICC > 0.3) for calm only. For Round Two, ICC ranged from 0.0983 (confused) to 0.361 (calm) with reliability being highest (ICC > 0.3) for agitated, calm, frightened and stressed. Between Round One and Round Two, ICCs were similar for anxious, calm, excited and exhausted.

### 3.4. ETA

The responses from Part 3 of the ETA survey (*n* = 16) were compared to the QBA scores for each participant. Observers who more strongly agreed with “I hate seeing pictures of animals used in scientific experiments” and “Seeing animals in pain upsets me” had significantly higher Principal Component Factor 1 scores for Chase A than those who less strongly agreed (*p* = 0.01, *p* = 0.03, respectively). Principal Component Factor 1 was mainly composed of agitated, anxious, stressed, frightened and confused.

Observers who more strongly agreed with “I get annoyed by dogs that howl and bark when they are left alone” and “Often cats will meow and pester for food even when they are not really hungry” revealed more differences in FTD scoring between chase and recovery phases than those who less strongly agreed (*p* = 0.02, *p* = 0.03, respectively).

### 3.5. Ethogram

Binary logistic regression modelling was used to determine the most prevalent behaviours displayed by the calves during chase and recovery stages. Behaviours that were significantly more common in the chase phase than in recovery were Legs Gallop (Likelihood Ratio χ^2^ = 22.206; *p* < 0.001) and Tail Held Rigid (LR χ^2^ = 6.585; *p* = 0.010). Behaviours that were significantly more common in the Recovery phase than in the Chase phase were Ears Axial (LR χ^2^ = 7.034; *p* = 0.008), Ears Forward (LR χ^2^ = 6.560; *p* = 0.010), Legs Slow Run (LR χ^2^ = 20.360; *p* < 0.001) and Legs Fast Walk (LR χ^2^ = 13.983; *p* < 0.001).

## 4. Discussion

The QBA scoring showed a clear pre- and post-rope effect on the emotional state of calves observed during the calf-roping event. Observers thought that calves during the chase phase were more agitated, anxious, confused, energetic, frightened and stressed. Agitation, anxiousness, fear and stress, as well as calves commonly holding their tails rigidly, are attributed to the calf being pursued by a rider on horse-back at speed, most likely simulating a predator/predation effect. Similarly, confusion scores may be a result of this predation effect, but could also be credited to calves struggling to take in as much information as possible about their surroundings and atmosphere at the time of release from the holding chute. The ethogram scores for the chase phase indicate that the calves were always galloping and may explain why observers viewed calves as being energetic.

In contrast, observers scored calves in the recovery phase as more calm, contented, exhausted, inquisitive and relieved than in the chase phase. Calmness, contentedness and relief were most likely a result of the rope being removed and no horse and rider chasing the calf at speed. The ethogram scores showed that calves were moving slower during the recovery phase than in the chase phase. This may help to explain why observers saw the calves as exhausted, as well as calm, contented and relieved. Linked to the higher scores for confused in the chase phase, inquisitiveness in the recovery phase was most likely a response to the calves assessing their surroundings, though this time in a more relaxed emotional state. Inquisitiveness was also suggested from the behavioural scoring of ear position being more commonly axial and forward, rather than pinned or facing backwards. These results indicate that the use of a QBA approach differentiated emotional states of calves between the chase phase and the recovery phase. In addition, observers identified specific emotional experiences expressed by calves during the chase phase that were characterised by more negative experiences than in the recovery phase. Although the 20 calves whose images were used were roped by different riders, it is possible that the skill and speed of competitors may influence the behaviour and emotional state of calves. To explore the effects of riders’ skill levels on calf welfare, future studies may factor-in the level of competition of the events in which the calves are being used.

The differences in emotional state during chase and recovery phases seen in this study suggest that roping events induce an acute stress response in calves. This supports recent research [1] where serum cortisol, epinephrine and nor-epinephrine concentrations of calves increased after being roped but were no longer significantly elevated 2 h post-roping. Given that these hormones are often recognised as indicators of stress, this would explain why scores for stressed and agitated were significantly lower in the recovery phase than the chase phase. Likewise, the change in pace of the calves between the phases, from galloping to slow running/walking, suggests an immediate change in emotional state. However, it should be noted that another study found that not only were cortisol concentrations increased in calves after the roping event, but they were further elevated at 24 h [10]. In that study, head-shaking and sham-chewing were evident in a third of the calves after they had been roped. There were significant increases in the plasma concentrations of creatinine kinase, lactate dehydrogenase enzymes and cortisol in roped calves. However, these increases could not be attributed solely to being roped, thrown and tied because significant increases were also found in calves who had been pursued by riders but not roped successfully. It is possible that physiological measures 24 h after roping may be unrelated to the specific intervention of current interest (i.e., roping) and could be compounded by marshalling or herding, transporting and blood sampling.

Human interaction may have also played a role in observed changes to the behavioural and emotional states of calves in this study. Calves appeared to be more calm, contented and relieved and moved slower when little human interaction was present; i.e., in the recovery phase when the rope had been released and the calf had been left alone to exit the arena. In contrast, calves were more stressed, frightened, anxious, agitated and moved faster when human interaction was intense; i.e., during the chase phase. Similar responses to human interaction at rodeos have been reported [2] in bucking bulls during marshalling and mounting phases of bucking bull events, where behaviours also likely to be associated with stress and anxiousness such as rearing and tail-swishing, were exhibited. Half of those who showed such signs did so in association with human interactions, such as when personnel were approaching or standing at the head of the bull or standing in the bull’s direct line of sight, or pre-empting release from the chute as riders were preparing to mount the bulls. None of these behaviours appeared to compromise the bucking performance of the animals. Given that this study revealed less conflict behaviour among the veteran animals, it may suggest that animals become habituated to events associated with rodeos. However, the authors acknowledged that they were unable to explore whether the lack of proactive conflict behavior in these animals was in itself a manifestation of learned helplessness, rather than habituation.

Observers in Round One, who had extensive cattle experience, showed less agreement than the students on how they scored each image. It may be that variation in level and type of experience contributes to how observers assess emotional states in animals. Over the course of their careers, practitioners have honed their observation skills in assessing the emotional and behavioural state of animals under veterinary care, which may be why the only term consistently scored by practitioners was calmness. A previous study [29] has found that veterinarians consistently identified procedures that were painful for animals, but it may be that seeing animals in states of pain, fear and stress over years of clinical practice could desensitise them to subtler changes in the emotional and behavioural states of calves used in the entertainment industry. This may contribute to the relatively low agreement across the other emotional descriptors.

Student observers showed higher agreement than practitioners in how they scored images. They agreed the most on calm, agitated, frightened and stressed. Compared to practitioners, current students are likely to be undergoing similar education, an attribute which could explain why they have higher agreement. Furthermore, students may not be desensitised to seeing animals undergoing emotional challenges. The current ETA data revealed that students who had more empathy for animals in pain and for those used in experiments were more empathetic towards calves during the chase phase. They felt that calves being chased were agitated, anxious, stressed, frightened and confused. To the authors’ knowledge, this is the first report of links between empathy towards animals and QBA scoring. As the ETA survey was not conducted with the practitioner group, the influence of attitudes to animals could not be evaluated in relation to the observation response scores for this group.

Despite there being low rate of agreement within both rounds as shown by the ICC scores, there was a moderate level of agreement between rounds as evident by the similarities in the Principle Component Analysis. Factor 1 was broadly similar in both rounds, Factor 2 in Round One resembled Factor 3 in Round 2, and vice versa. This implies that scoring within both groups was influenced by the same groupings of emotions, whether they were negative, positive or neutral emotions. Unfortunately, due to the limited number of observers, it was not possible to compare how the ICC of responses within the veterinary/animal science student group changed in association with their attitudes to animals. Future studies to explore this relationship would benefit by having sufficient a number of observers

One limitation of the current study was the use of still images as, generally, QBA studies use video/film footage for FTD scoring. This may have contributed to lower scores of agreement as video footage provides a more holistic view of the animals’ emotional state and provides context as to the animal’s surroundings within that space of time. In any attempt to identify emotional state, the use of only two still images depicting each of the chase or recovery phases might be considered brief. However, given the rapid speed of calf roping events, it is reasonable to assume that these still images are representative; i.e., on average, the chase phase lasts less than four seconds. Also, observers were blinded to the nature of the event occurring. Furthermore, still images have been used in other studies to detect the presence of pain in horses [33,34]. Also, it should be noted, compared to video footage, that still images are less likely to capture all aspects of body language. Thus, the quality of the QBA scoring for still images may not be as high as for video footage. 

Another potential limitation was the difference in sample size between Round One and Round Two. The smaller sample size of Round One compared to Round Two may contribute to less accurate ICC scores. It should also be noted that there was some variability in the level of experience in the observer groups. Lastly, recruitment of participants from a limited pool of students was countered by the use of the empathy survey. By taking into account how observers viewed their own level of empathy towards animals, it enabled links to be drawn between how a wider audience with different levels of empathy towards animals may assess animal emotional states. 

Rather than using still images, further QBA studies in this area should use footage of calves during roping events. Rodeo-specific elements in these videos, such as background features, ropes, rodeo horses and competitors could be obscured; e.g., by pixelation. Future research could also aim to explore associations between perceived emotional state during chase and recovery phases with physiological measures.

## 5. Conclusions

QBA of still images has the potential to be an assessment tool for animal welfare in the entertainment industry. The current ATA data revealed that students who had more empathy for animals in pain and for those used in experiments were more empathetic towards calves during the chase phase. They felt that calves being chased were agitated, anxious, stressed, frightened and confused. Both experienced practitioners and veterinary and animal science students successfully identified differences in the emotional state of calves during the chase and recovery phases of roping events. Observers generally saw calves during the chase phase as being relatively agitated, anxious, confused, energetic, frightened and stressed. In contrast, they saw calves in the recovery phase as calm, contented, exhausted, inquisitive and relieved. These findings indicate that calves in roping events experience several negative emotions, which raise serious concerns as to the continuation of these events on welfare grounds.

## Figures and Tables

**Table 1:** Factor loadings of Factor Analysis of Fixed Term Descriptors applied by a panel of seven practitioners on 40 images of calves (*n* = 10) during a calf roping event.

Descriptor	Factor1	Factor2	Factor3	Factor4
Agitated	0.8			
Anxious	0.84		−0.32	
Confused	0.69			
Frightened	0.91			
Stressed	0.87			
Energetic		0.93		−0.34
Excited		0.84		
Inquisitive		0.51	0.47	
Calm			0.87	
Contented			0.8	
Relieved			0.44	0.53
Exhausted	0.41	−0.36		

**Table 2:** Factor loadings of Factor Analysis of Fixed Term Descriptors applied by a panel of 16 students on 80 images of calves (*n* = 20) during a calf roping event.

Descriptors	Factor1	Factor2	Factor3	Factor4
Agitated	0.82			
Anxious	0.91			
Confused	0.62			
Frightened	0.9			
Stressed	0.92			
Calm		0.8		
Contented		0.95		
Relieved		0.57	0.3	
Energetic			0.77	
Excited			0.84	
Inquisitive		0.41	0.55	
Exhausted	0.34			0.54

**Table 3:** Comparison between Factor Analysis for Fixed Term Descriptors applied by a panel of 16 students on 80 images of calves (*n* = 20) during the calf pursuit (chase) and calf recovery after roping (recovery) phases of a calf roping event. Significant values appear in bold.

Phase	Factor 1			Factor 2			Factor 3			Factor 4		
	Coeff	SE	*p*-Value	Coeff	SE	*p*-Value	Coeff	SE	*p*-Value	Coeff	SE	*p*-Value
Chase B	−0.071	0.074	0.340	0.001	0.075	0.993	0.192	0.071	**0.007**	0.000	0.054	1.000
Recovery C	−0.498	0.074	**<0.001**	0.404	0.075	**<0.001**	−0.206	0.071	**0.004**	0.286	0.054	**<0.001**
Recovery D	−0.640	0.074	**<0.001**	0.708	0.075	**<0.001**	−0.141	0.071	**0.046**	0.300	0.054	**<0.001**

**Table 4:** Intra-Class Correlation Coefficients for Round One (seven practitioners on 40 images of calves) and Round Two scoring (16 students on 80 images of calves) of fixed term descriptors of calves in a calf roping event.

Descriptors	ICC Round One	ICC Round Two
Agitated	0.127	0.303
Anxious	0.176	0.244
Calm	0.306	0.361
Confused	−0.00775	0.0983
Contented	0.139	0.292
Energetic	0.104	0.209
Excited	0.134	0.179
Exhausted	0.200	0.176
Frightened	0.174	0.320
Inquisitive	−0.0307	0.184
Relieved	−0.0378	0.126
Stressed	0.0658	0.312
